# Safety of cardiorespiratory and muscle fitness assessment in two children with leukemia during early chemotherapy

**DOI:** 10.1177/18758894251347128

**Published:** 2025-06-27

**Authors:** Virginie Aspirot-Buron, Charles Sèbiyo Batcho, Michèle Bisson, Bruno Michon, Marc-André Dugas, Isabelle Marc, Philippe Corbeil

**Affiliations:** 1 Department of kinesiology, Université Laval, Quebec city, Quebec, Canada; 2 Centre interdisciplinaire de recherche en réadaptation et intégration sociale (Cirris), Quebec city, Quebec, Canada; 3 Department of rehabilitation, Université Laval, Quebec city, Quebec, Canada; 4 Faculty of Medicine, Université Laval, Quebec city, Quebec, Canada; 5 Centre hospitalier universitaire de Quebec, Quebec city, Quebec, Canada

**Keywords:** childhood cancer, hematologic cancer, physical status, cardiorespiratory, physical activity

## Abstract

**Purpose:**

Limited data are available on the cardiorespiratory and muscle fitness of children with acute lymphoblastic leukemia (ALL) during chemotherapy. This pilot study evaluated the safety of testing the cardiorespiratory and muscle fitness of two children with ALL at different risk levels in early chem treatment.

**Methods:**

Two girls with low- and high-risk B-cell ALL (DFCI-16-001) took part in two test sessions: T1 (induction, consolidation 1A/C) and T2 (consolidation 2). Each testing session included a maximal oxygen uptake ( $\dot{V}$ O_2_max) exercise test, muscular strength tests, physical activity and quality of life questionnaires, and a semi-structured interview. The parents agreed to these assessments at the start of chemotherapy treatment.

**Results:**

The participants experienced no significant adverse effects from undertaking the cardiorespiratory and muscular tests, and there was no impact on their chemotherapy treatment schedule. At their post-test interview, both participants reported that thigh pain and fatigue were the most difficult part of the $\dot{V}$ O_2_max exercise test. Regarding physical performance outcomes, both participants exhibited low scores compared to their gender, weight- and age-predicted $\dot{V}$ O_2_max and on most strength test values.

**Conclusion:**

The physical tests were safely and successfully conducted with these two participants during early chemotherapy.

## Introduction

Acute lymphoblastic leukemia (ALL) is the most common type of childhood cancer. In Canada, from 2009 to 2013, it represented approximately 32% of all cancer diagnoses. ALL is the most prevalent type of leukemia, accounting for approximately 80% of cases.^1^ From 2000 to 2004, the five-year survival rate was 91%_._^2^ However, chemotherapy has deleterious consequences for children's physical fitness. Certain chemotherapeutic drugs such as vincristine, dexamethasone and doxorubicin have been shown to negatively impact muscle function, strength and integrity in the upper and lower limbs, as well as the heart.^3^ Chemotherapy can cause neuropathies, muscle atrophy and a decrease in cardiorespiratory fitness.^4–7^ Strong chemotherapeutic drugs, such as anthracyclines, can cause cardiac toxicity in long-term ALL survivors (15 years post-diagnosis), resulting in lower cardiorespiratory capacity than in adults who are not cancer survivors.^4,5^ In the worst-case scenario, this cardiotoxicity may lead to heart failure.^8^ The minimum cumulative dose at which the incidence of chronic cardiac toxicity increases significantly and the exact mechanism of development of this serious complication are still investigated.^9–11^

Typically, chemotherapy includes three main phases: induction followed by consolidation and continuation (also known as maintenance). The induction phase is characterized by the highest dosage of chemotherapeutic agents most often including vincristine, anthracyclines and glucocorticosteroids, and aims to destroy as many leukemic blasts as possible. A study by Ness et al., which indirectly measured the cardiorespiratory capacity of children with ALL using a modified six-minute walk test, reported that their capacity was already below normative values seven to ten days into induction chemotherapy. They also reported that their participants had significantly decreased passive ankle dorsiflexion and knee extension strength, which was associated with poor health-related quality of life in children, as per the physical function subscale of the questionnaire.^12^ Vriens et al. obtained similar results, namely an early decline in cardiorespiratory fitness, also measured by the six-minute walk test.^13^ They also found that these lower-than-normative values persisted throughout chemotherapy treatment and even six months post treatment.^13^ During the last phase of chemotherapy (maintenance), another study showed that children with ALL had lower muscle strength than age- and sex-matched comparison groups.^14^

Several studies showed that side effects including chemotherapy-induced peripheral neuropathy and cardiotoxicity are correlated to cumulative dosage of chemotherapeutic drugs over time.^3,15,16^ Furthermore, cardiorespiratory fitness and muscle function are adversely affected in long-term ALL survivors due to the cumulative dosage of chemotherapeutic drugs.^3–5,8,10^ The onset of this deconditioning in the short term, i.e., early on in treatment of ALL, is of interest. Several authors have mentioned the difficulty of performing a substantial battery of tests in vulnerable young patients.^12,17,18^ A recent study showed that survivors of childhood ALL could safely take part in a maximal oxygen consumption test ( $\dot{V}$ O_2peak_).^19^ It has been proposed that such tests should be part of this population's long term cardiac care follow-up to identify any cardiac troubles and to make sure that physical activity interventions for these survivors are built upon their actual physiological capacities and are challenging enough.^19,20^ Furthermore, measuring the maximal oxygen uptake via direct measurement of gas exchange, heart rate and blood pressure, and measuring muscle function at different times during chemotherapy may lead to a better understanding of the physiological status of children with ALL and allow for adequate supervised and adapted cardiovascular and muscle training. To the authors’ knowledge, very few studies have reported cases of children performing a maximal exercise test a few weeks after completing the induction phase of chemotherapy.

The primary objective of this pilot study was to evaluate the safety of pediatric patients undergoing rigorous physiological testing during the first 10 months of chemotherapy to treat ALL, specifically cardiorespiratory and muscle function testing.

The secondary objectives were:

to assess participant and family perception of testing difficulty and to assess their interest in participating in a larger-scale intervention involving physical activity.
to collect, interpret and evaluate the accuracy and validity of the data from the cardiorespiratory and muscular strength tests in order to better understand the physiological changes the participants experienced between the testing sessions.

A mixed methods design, i.e., a one phase design in which both quantitative and qualitative data were collected and analyzed, was conducted to answer the objectives.

## Methods

### Experimental approach to the problem

This pilot study was conducted in the Reproduction, Mother and Youth Health Department at the Centre mère-enfant Soleil of CHU de Québec-Université Laval*.* Eligible participants were treated solely with chemotherapy throughout the different phases of their treatment protocol. Children who received cranial radiation therapy or a bone marrow transplant as their treatment regimen were excluded from this study. Those with Down syndrome were excluded due to their variance in genetics. Children with a congenital heart anomaly revealed by a pre-chemotherapy echocardiogram and those with a history of musculoskeletal condition that would preclude them from engaging in strenuous exercise were also excluded.

A mixed method of data collection was used in this pilot study. The data was obtained through cardiorespiratory fitness and muscle strength testing, physical activity and quality of life questionnaires, semi-structured interviews conducted with the participants and their parents, and anthropometric measurements. Cardiorespiratory fitness was assessed through maximal oxygen uptake ( $\dot{V}$ O_2_max) as this is the gold standard.^21,22^ Muscle strength was assessed through hand-held dynamometry in order to evaluate if the muscular strength of these children with ALL undergoing chemotherapy would develop as they aged, as suggested by the normative values of Hebert et al.^23^

### Participants

Of the five eligible children with ALL and their families who were approached for this pilot study, two accepted to participate and three declined.

**Figure 1.**
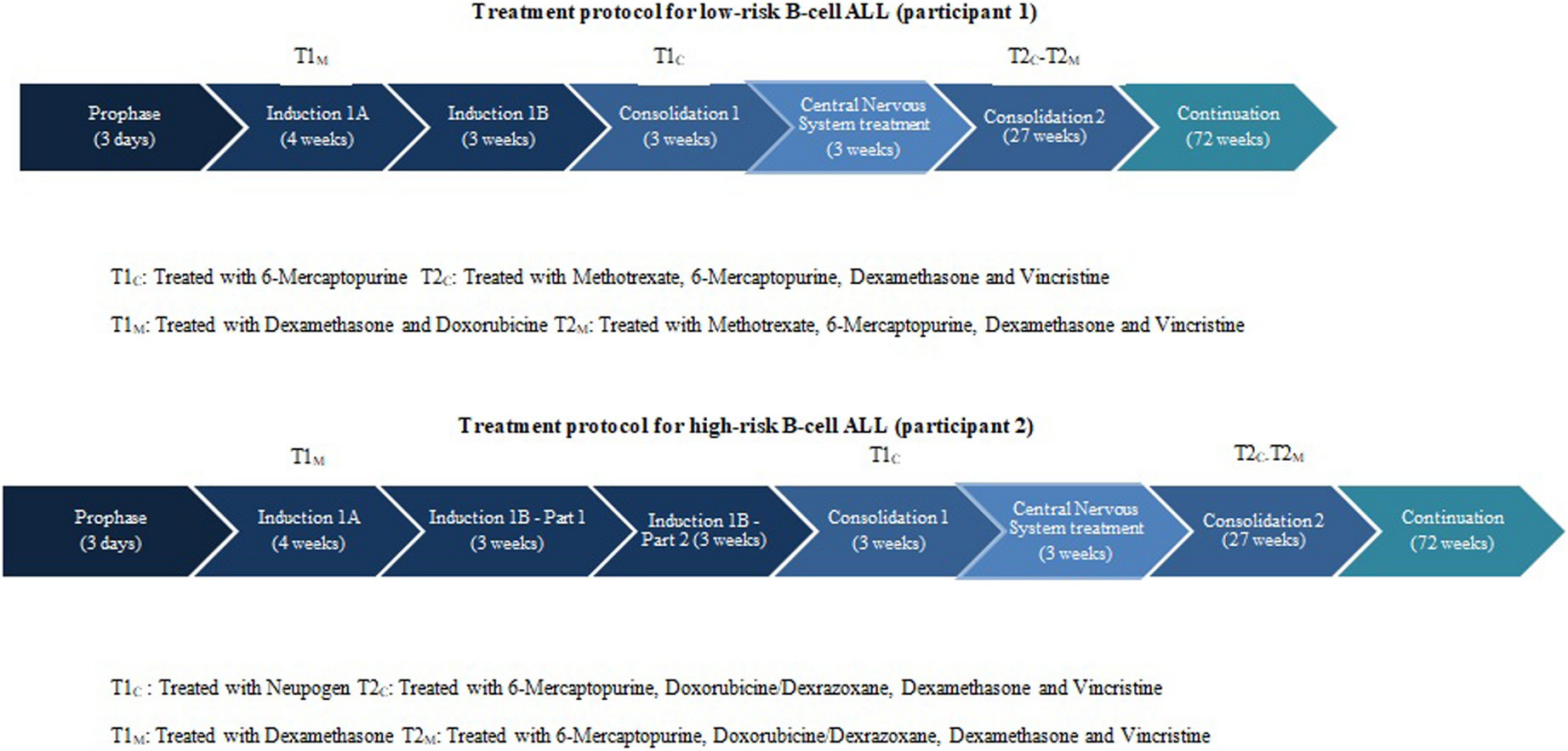
Treatment protocol for low- and high-risk B-cell acute lymphoblastic leukemia (ALL) administered to the participants and their cardiopulmonary (T1-2C) and muscular (T1-2 M) testing time points. Chemotherapeutic agents that were prescribed at these times are also indicated.

The two children who took part in this pilot study were an 11-year-old female with a diagnosis of low-risk B-cell ALL (participant 1, weight: 48.0 kg; height: 163.3 cm) and a 12-year-old female with an initial diagnosis of low-risk B-cell ALL, who became high-risk during the course of the study (participant 2, weight: 62,2 kg; height: 166.3 cm). They were treated at the Centre mère-enfant Soleil of CHU de Québec-Université Laval under the DFCI-16-001 protocol (Figure 1). The participants had periodic follow-ups with their medical team.

The study was reviewed and approved by the hospital's Ethics and Committee Review Board (2018–3727). In addition to written informed consent from the parents and positive assent from the participants, medical approval was obtained before each test session to ensure the participants’ safety.

### Procedures

#### Level of physical activity and quality of life questionnaires

Questionnaires were administered to the participants and their parents to assess physical activity level, using a French translation of the Habitual Activity Estimation Scale (HAES).^24^ This qualitative and quantitative questionnaire yielded data on the participants’ daily routine as well as the days of the week, and on the moments during a day when the participants were the most and least physically active. The quality of life questionnaire administered to both the participants and their parents was the PedsQL Cancer Module 3.0.^25^ It yielded an overall score of the participants’ perceived quality of life based on their scoring of cancer-related elements such as pain and hurt, nausea, and anxiety about the procedures. A lower score on a scale of 1–100 indicated poorer quality of life. The data collected from both the participants and their parents allowed for a comparison between the participants’ perception of their physical activity level and quality of life versus their parents’ perception.

#### Cardiorespiratory fitness testing

The first cardiorespiratory fitness test session was during the second week of consolidation 1A on the 62^nd^ day after diagnosis for participant 1 and on the 132^nd^ day, in consolidation 1C, for participant 2. The second test session (T2) was in the consolidation 2 phase of chemotherapy on the 167^th^ day after diagnosis for participant 1 and the 301^st^ day for participant 2 (Figure 1).

Maximal oxygen uptake ( $\dot{V}$ O_2_max) was assessed with the James protocol^21^ by a Certified Exercise Physiologist using a multistage incremental maximal exercise test on a cycle ergometer. It was equipped with vertically adjusted handles for comfort, so that the participants’ elbows were slightly bent and their shoulders relaxed. The seat height was also adjusted vertically so the knees were slightly flexed at full extension. For safety reasons, the ergometer had adaptable pedal grips to secure the feet. Following the American Heart Association's recommendations,^26^ participants’ gas exchanges (Ultima Cardio2, MCG Diagnostics, MN), heart rate with a 12-lead electrocardiogram, and pulse oximetry with a finger sensor (Nonin 7500, Nonin Medical Inc., MN) on the index finger of the left hand were continuously measured throughout the test. Blood pressure was measured with an automated cuff (TANGO+, SunTech Medical Inc., NC) during a 1 min warm-up period, every 2 min during the test, and at the end of the active cooldown. Each stage lasted 3 min and the test continued until the participants could no longer continue due to volitional fatigue. Performed $\dot{V}$ O_2_max was calculated by graphing oxygen consumption. Secondary criteria for determining if a maximal effort was achieved during the test included a respiratory exchange ratio (RER) of > 1.1 and a heart rate within 10% of age-predicted maximal heart rate. Values of $\dot{V}$ O_2_max as shown in Table 1 were calculated both as an absolute rate (ml/min) and in relation to body weight (ml/kg/min). The formula used to predict the maximal heart rate was 220 - Age.^27^ The formula used to calculate body surface area was 0.024265 * (Height * 100) ^0.3964^ * Weight ^0.5378^.^28^ Two formulas were used to predict $\dot{V}$ O_2_max: if body surface area < 1.2, then $\dot{V}$ O_2_max =185.72 + 2.23 * (928 * Height - 780); if body surface area > 1.2, then $\dot{V}$ O_2_max =185.72 + 2.23 * (2065 * Height) - 2484)^29^; where body surface area, weight and height are expressed in m^2^, kg and m, respectively.

**Table 1.** Anthropometric characteristics, $\dot{V}$ O_2_max and muscle strength tests results for participant 1 and participant 2 at post-induction (T1) and during consolidation 2 (T2).

Anthropometrics	Participant 1			Participant 2		
	**T1** (62 days after diagnosis)	**T2** (167 days after diagnosis)	**Δ (%) between T1 and T2**	**T1** (132 days after diagnosis)	**T2** (301 days after diagnosis)	**Δ (%) between T1 and T2**
Age at test session (years)	11	12		12	13	
Weight (kg)	48.0	53.9	↑ 12.3	62.2	70.4	↑ 13.2
Body surface area (m²)	1.47	1.57	↑ 6.8	1.7	1.8	↑ 5.9
BMI (kg/m²)	18.0	19.9	↑ 10.6	22.5	25.4	↑ 12.9
Body fat (%)	21.6	28.6	↑ 32.4	29.9	43.5	↑ 45.5
Lean body mass of left leg (kg)	5.95	6.17	↑ 3.7	6.53	6.57	↑ 0.6
Lean body mass of right leg (kg)	5.89	6.08	↑ 3.2	6.56	6.62	↑ 0.9
Muscle strength tests*						
Grip strength of left arm (Kgf)	11	14.5	↑31.8	16.2	21.5	↑ 32.7
Grip strength of right arm (Kgf)**	17	16.5	↓2.9	16.5	25.5	↑ 54.5
Moment of force of left knee extensor (Nm)	30.1	56.8	↑ 88.7	75.4	75.6	↑ 0.3
Moment of force of right knee extensor (Nm)	28.3	63	↑ 122.6	58.9	81.1	↑ 37.7
Moment of force of left ankle dorsiflexor (Nm)	5.8	7.2	↑ 24.1	12.2	8.9	↓ 27.0
Moment of force of right ankle dorsiflexor (Nm)	5.5	8.2	↑ 49.1	10.2	8.5	↓ 16.7
**Maximal exercise test**						
Total exercise time (s)	361	442	↑ 22.4	599	429	↓ 28.4
$\dot{V}$ O_2_max (ml/min)	884.6	1366.9	↑ 54.5	1472.5	1216.7	↓ 17.7
% of predicted $\dot{V}$ O_2_max	41.1	60.9		64.3	53.1	
$\dot{V}$ O_2_max (ml/kg/min)	18.4	25.4	↑ 38.0	23.7	17.3	↓ 27.0
% of predicted $\dot{V}$ O_2_max	44.8	41.7		64.3	53.2	
Maximum heart rate (bpm)	169.4	175.0	↑ 3.3	184.7	199.7	↑ 8.1
Maximum systolic blood pressure (mmHg)	125.9	150	↑ 19.1	174	167.1	↓ 4.0
Minute ventilation (VE) BTSP^1^ (L/min)	37.1	54.0	↑ 45.6	55.1	63.3	↑ 14.8
% Maximum voluntary ventilation	34.0	48.2	↑ 41.8	49.2	49.8	↑ 1.2
Respiratory exchange ratio	1.50	1.26	↓ 16.0	1.2	1.4	↑ 11.2

*The T1 grip strength test for participant 1 was done 12 days after diagnosis, and the lower limb tests were done 26 days after diagnosis (Induction 1A). All tests for participant 1 at T2 were done 170 days after diagnosis (Consolidation 2). For participant 2, T1 tests were done 14 days after diagnosis (Induction 1A) and T2 tests 176 days after diagnosis (Consolidation 2).^1^ BTSP: body temperature and ambient pressure saturated with water vapor. ** For both participants, the right hand was dominant.

For both testing sessions, four different measurement periods (M1 to M4) were analyzed: M1: the last 30 s (s) of the warm-up period; M2: the last 30 s of stage 1; M3: the last 30 s of the first test (T1), just before the cooldown period; M4: the last 30 s of the overall test just before cooldown. The M3 measurement was designed so that the physiological measurements could be compared at the same time point (e.g., from the 5 min mark to 5 min 30 s mark) on both tests if a participant did better on their second testing session (T2) than on their first (T1). Participants were asked to rate their perceived exertion on the Borg scale^30^ every 2 min from the warm-up to the cooldown. A physician was either present in the laboratory or immediately available on-call during the testing sessions.

Anthropometric measurements, such as height (EZ-Glide 84”, Hopkins Medical Products, MD), body weight, lean body mass, body fat and body mass index (InBody 520, InBody Co. Ltd, CA) were collected at both testing sessions.

#### Muscular strength testing

Grip strength (JAMAR Technologies, PA) and knee extensor and ankle dorsiflexor moment of force were assessed with a hand-held dynamometer (Lafayette Instrument Co., IN).^31^ Grip strength was tested with the participant in the upright position, with shoulder, elbow and wrist held alongside the body. Knee extensor moment of force was assessed with the participants seated on an anti-slip mat, trunk upright, and hips and knees flexed at 90°. Participants were allowed to hold on with their hands during the lower-limb exertion. Ankle dorsiflexor moment of force was assessed with participants lying supine on an anti-slip mat with their ankles flexed at 90° and feet unsupported. Strength tests were performed on both sides. Participants performed two familiarization trials at around 50% of maximal effort before they performed two attempts at maximal effort. Participants were encouraged to gradually produce maximum force and hold it for 3–5 s before releasing. The mean value of the two trials was retained for analysis. Verbal encouragement was given by the evaluator throughout the attempt to encourage participants to give maximal effort on each attempt. For participant 1, grip strength was assessed on the 12^th^ day after diagnosis and lower limb testing was done on the 26^th^ day after diagnosis in induction 1A for T1 (Figure 1). At T2, all tests were done on the 154^th^ day after diagnosis in consolidation 2. For participant 2, all tests were conducted in induction 1A on the 14^th^ day after diagnosis for T1 and on the 176^th^ day after diagnosis in consolidation 2 for T2 (Figure 1).

#### Perceived difficulties and interest in participating in a physical activity intervention

After both cardiorespiratory fitness testing sessions, the experimenter conducted two short semi-structured interviews, one with the participant and one with the parents (see Appendix A for the questions on the semi-structured interview). The participants were asked to rate their perception of the test's level of difficulty, identify what was the most difficult element of the test (e.g., shortness of breath, muscle cramps, pain in the legs/thighs), and say whether or not they engaged in physical activity at home and if they would be interested in participating in group training with other children with ALL also undergoing chemotherapy treatment. The parents were asked similar questions on their perception on the test's level of difficulty and on their child's performance during the test, if they did any physical exercises as family activities and if they would be interested in having their child participate in group training with other children with ALL.

#### Test safety evaluation

Occurrence of any adverse effects related to the cardiorespiratory and muscular strength test that had an impact of the participants’ physiological state, treatment regimen or schedule were noted.

### Data interpretation

Results from the qualitative data are expressed as aggregate or as individual responses from the questionnaires administered and the semi-structured interviews conducted with the participants and their parents.

For the quantitative data, values are expressed as relative percentage difference between both testing sessions ((T2-T1)*100/T1). The participants’ $\dot{V}$ O_2_max values were compared to the normative values calculated online by BreezeSuite software v. 7.2.0.64 (Medical Graphics, MN). Grip strength and moment of force for the lower limbs were compared to standard reference values with standard deviation.^23,32^

## Results

### Participant 1

During the semi-structured interview, for both T1 and T2, participant 1 said that she found the cardiorespiratory fitness test's level of difficulty as medium (choices were easy, medium or hard). For both T1 and T2, the participant identified that the most draining part of the test was related to fatigue in her thigh muscles. However, at T2, she found the test easier to do because she considered herself in better overall shape. Her parent also mentioned that they perceived that their child had done better on the test because they perceived her to be in better shape and to have increased her energy level. The participant and the parent both reported that she was engaging regularly in physical activity at T1 and T2. Some activities were done alone (e.g., elliptical) and some through family activities (e.g., badminton, cycling, snowshoeing, hiking and stair climbing).

At T1, the participant reported being slightly less active than three months before, during both her typical weekday and on Saturday. At T2, she reported spending 39.6% of her time during weekdays engaged in vigorous activities compared to none at T1. A similar increase was also reported by her parent.

From T1 to T2, her quality of life score increased by 35.2% from 71 to 96, according to the participant, and by 34.9% from 63 to 85, according to her parent; both reported decreased frequency of joint and muscle pain.

From T1 to T2, participant 1 had experienced a 12.3% weight gain, an 8.2% loss of lean body mass and a 32.4% increase in body fat. She had a 3.7% and 3.2% increase in lean body mass for the left and right leg, respectively (Table 1).

Also, from T1 to T2, there was a 31.8% improvement in her grip strength for the left hand and a 2.9% loss for the right hand. Maximum knee extensor and ankle dorsiflexor moment of force increased from T1 to T2 for the right and left limbs (Table 1). According to the normative values in Hebert et al. adjusted for the participant's age, her percentile ranks ranged from 2^nd^ to 10^th^ for the moment of force for her knee extensor, and from 4^th^ to 9^th^ for the moment of force of the ankle dorsiflexor, for the left and right sides. All strength values at both times were lower than those of healthy children matched for age and gender.^23^

**Figure 2.**
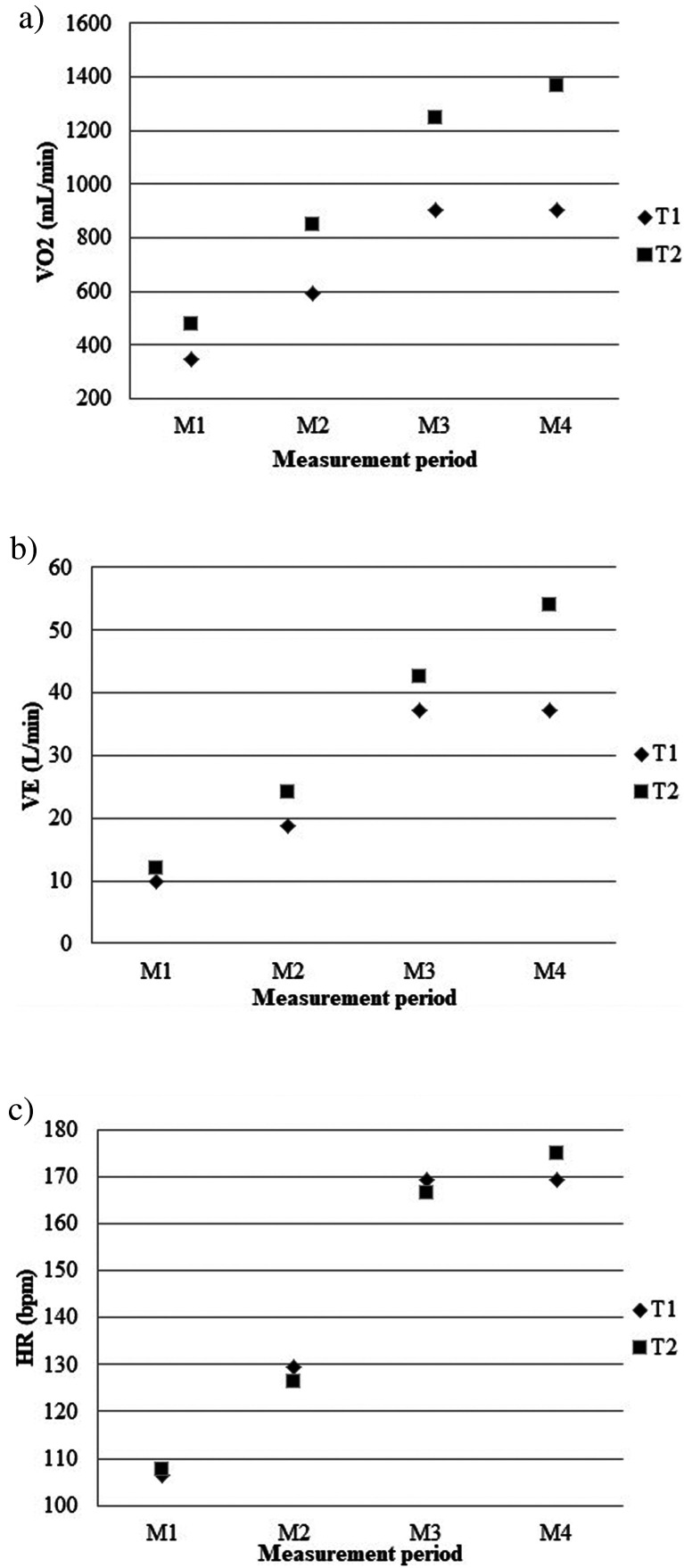
Physiological parameters of participant 1 during the cardiorespiratory tests at T1 and T2. a: minute ventilation (l/min); b: heart rate (HR; measured in beats per minute [bpm]); c: oxygen consumption (ml/min); M1: the last 30 s (s) of the warm-up period; M2: the last 30 s of stage 1; M3: the last 30 s of the first test (T1), just before the cooldown period; M4: the last 30 s of test overall test just before cooldown.

Figure 2 shows oxygen uptake ( $\dot{V}$ O_2_), minute ventilation and heart rate at four measurement periods: M1-M4. The test duration was 6 min 1 s at T1 and 7 min 22 s at T2. From T1 to T2, participant 1 experienced a 45.6% increase in minute ventilation and a 16.0% decline in RER (Table 1). Her peripheral saturation of oxygen was above 97% throughout both tests. For cardiac function, her maximum heart rate achieved during the test improved by 3.3% and her maximum systolic blood pressure by 19.1% (Figure 2). $\dot{V}$ O_2_max (ml/min) increased by 54.5% (Figure 1) from T1 to T2 (Table 1) but remained lower than the expected values for healthy children matched for age, gender and weight.

### Participant 2

During the semi-structured interview, the participant said that she found the test's level of difficulty as medium at both T1 and T2 (choices were easy, medium or hard). Her parents also perceived the test's level of difficulty as medium at both sessions. For both T1 and T2, the participant identified that the most draining part of the cardiorespiratory fitness test was related to pain in both of her thigh muscles. At T2, the participant found the test harder to do because of injections administered to treat neuropathic pains in her legs. Her parents mentioned that they perceived that their child had done well on the test, and they said that they thought that their child's energy level was slightly better than the previous test. The participant and the parent both reported that the participant was not engaging in any regular physical activity at T1 and T2.

At T1, the participant reported being slightly less active than three months before, during both her typical weekday and on Saturday, while her parents reported that she was much less active. At T2, both the participant and her parents reported that she was much less active than three months before. At T1, she reported spending 12.6% of her time during weekdays engaged in vigorous activities compared to none at T2. At T1 and T2, her parents reported that their daughter did not engage in any vigorous activities during the week or on Saturdays.

From T1 to T2, her quality of life score decreased by 20% according to the participant, and 26% according to her parents. The participant reported an increased frequency of problems with at least one of the measured indicators in each of the following main categories: pain and hurt, nausea, anxiety about the procedure, treatment anxiety, worry, cognitive problems and communication. The parents reported a similar increase in frequency except for communication, which stayed the same. In addition, they reported an increase in the frequency of problems in the perceived physical appearance category.

From T1 to T2, the participant experienced a 13.2% weight gain, an 8.7% loss of lean body mass and a 45.5% increase in body fat. Lean body mass for the left and right leg increased by 0.6% and 0.9%, respectively (Table 1).

There was a 32.7% and 54.5% improvement in her grip strength for the left hand and right hand, respectively, from T1 to T2. Maximum moment of force of the knee extensor increased bilaterally while the maximum moment of force of the ankle dorsiflexor decreased bilaterally from T1 to T2 (Table 1).

According to normative values^23^ adjusted for age, the participant's percentile ranks ranged from 24^th^ to 54^th^ for the moment of force of the left and right knee extensor and from 9^th^ to 98^th^ for the moment of force of the ankle dorsiflexor for the left and right sides. The moment of force of the right knee extensor at T1 and of both ankle dorsiflexor at T2 were lower than those of healthy children matched for age and gender.

**Figure 3.**
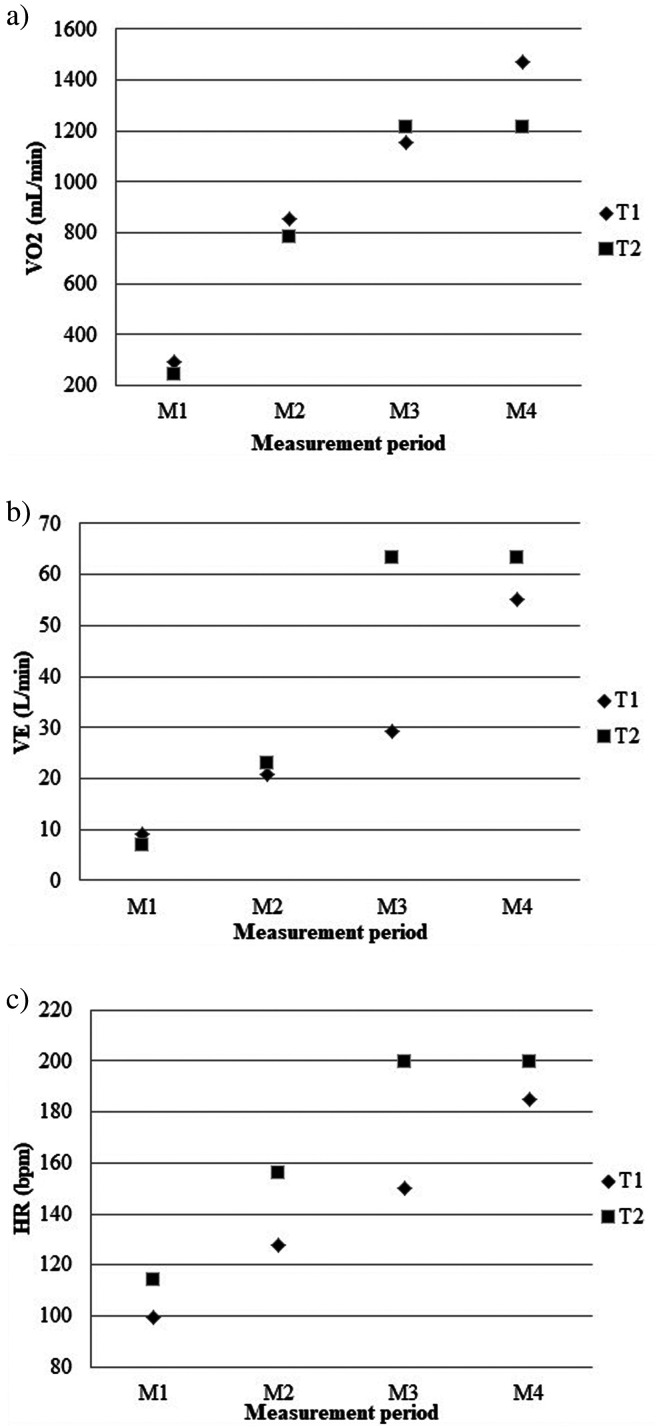
Physiological parameters of participant 2 during the cardiorespiratory tests at T1 and T2. a: minute ventilation (l/min); b: heart rate (HR; measured in beats per minute [bpm]); c: oxygen consumption (ml/min); M1: the last 30 s (s) of the warm-up period; M2: the last 30 s of stage 1; M3: the last 30 s of the first test (T1), just before the cooldown period; M4: the last 30 s of test overall test just before cooldown.

Figure 3 shows oxygen uptake ( $\dot{V}$ O_2_), minute ventilation and heart rate at four measurement periods (M1–M4) at T1 and T2. The test duration was 9 min 59 s at T1 and 7 min 9 s at T2. From T1 to T2, participant 2 experienced a 14.8% increase in minute ventilation and an 11.2% increase in RER (Table 1). Her peripheral saturation of oxygen was above 97% throughout both tests. For cardiac function, the maximum heart rate she achieved increased by 8.1% and her maximum systolic blood pressure decreased by 4.0% (Figure 3). $\dot{V}$ O_2_max (ml/min) decreased by 17.7% (Figure 2), from T1 to T2 (Table 1). Both of her $\dot{V}$ O_2_max (ml/min) scores were lower than the expected values for healthy children matched for age, gender and weight.

### Participants 1 and 2

No occurrence of serious adverse physiological symptoms or disturbance of the chemotherapy treatment regimen or schedule related to the cardiorespiratory and muscular fitness tests were reported for the two participants.

At T1, participant 1 reported enjoying playing a sport while participant 2 reported slightly enjoying physical activity. Both participants reported that they would not be interested in participating in adapted and supervised physical activity with other children with ALL undergoing chemotherapy treatment if it was offered to them. Both participants’ parents reported not seeing the interest of such group for their respective child either.

## Discussion

The primary objective of this pilot study was to evaluate the safety of testing the cardiorespiratory and muscle function of two young females with B-cell ALL undergoing the induction and consolidation phases of chemotherapy treatment under the DFCI-16-001 protocol. Of note, both participants successfully completed the battery of tests without any deleterious physiological consequences or negative impacts on their chemotherapy treatment regimen and schedule. Both participants were able to provide sustained cardiorespiratory effort as early as consolidation 1A and muscular effort as early as induction 1A (post induction) safely. Shortness of breath, volitional fatigue and a burning sensation in their thighs were also observed at the end of both tests.

While $\dot{V}$ O_2_max was calculated, only one of the secondary criteria (RER >1.1) was achieved in both test sessions for both participants. They did not reach a maximal heat rate of 200 bpm, which is an indicator of maximal effort.^26^ However, it is acknowledged that this threshold may not be reached in children with limitations to exercise^21,26^ such as in the case of cancer and a sedentary lifestyle during the first phase of chemotherapy.^18,33^ Participants’ willingness to exercise to the maximum of their ability may have been another factor limiting the achievement of a true measurement of maximal oxygen uptake ( $\dot{V}$ O_2_max). In this regard, the perception of testing difficulty was useful information for judging the degree of involvement perceived during the test, and both rated the level of difficulty of the cardiorespiratory as medium (from easy, medium or hard). Nevertheless, it is noteworthy that those in this pilot study were able to safely sustain a strenuous cardiovascular exercise for many minutes in order to perform a valid $\dot{V}$ O_2_ test post induction chemotherapy. This result is of importance because it suggests that it might be possible to safely collect a series of physiological data using evidence-based tests from the early stages of chemotherapy treatment, rather than waiting until the continuation phase, as has been done in previous studies.^6,34^

This finding is in agreement with a recent study in which survivors of childhood ALL with prior exposure to chemotherapy could safely take part in a maximal oxygen consumption test ( $\dot{V}$ O_2peak_).^19^ The study showed that participants (*n* = 216) completed a maximal cardiopulmonary exercise test confirmed by the achievement of two out of three of the criteria (RER value of ≥ 1.15, rate of perceived exertion score > 7, maximal heart rate ≥ 85% of the predicted value) with very few non-fatal adverse events at the end of the test and during the recovery period. In a multicenter non-randomized controlled trial, children with cancer, including ALL, and cancer-like disease treated with chemotherapy and/or radiation therapy participated in an intra-hospital physical activity program (including cardiorespiratory fitness, muscle strength and balance exercise) five days a week from diagnosis throughout intensive treatment.^35^ The researchers specified that they had not defined a targeted intensity for the program and that the goal was for the children to be as active as possible on any given training day. One year post-treatment, cardiorespiratory testing ( $\dot{V}$ O_2peak_) and handgrip strength tended to be higher in the intervention group than in the patient control group with cancer.^35^ Other studies, such as that by Fridh et al.,^35^ have shown that physiological testing can be attempted in early chemotherapy treatment to tailor early intervention programs to the physiological capacities of each participant. Several authors have proposed that such tests should be part of this population's long term cardiac care.^19,20^ This proposition is also supported by other researchers working with children with leukemia, who have suggested that it is important to implement physical activity at appropriate levels based on disease intensity and treatment phase.^36^

Perception of the participants in their ability to engage in physical activity was positive overall. The participants and their parents stated that they would not be interested in a supervised adapted physical activity with other children with ALL during their chemotherapy treatment, if this were offered to them. This result was observed in two families with opposite physical activity habits for the duration of study. Indeed, the parents of participant 1 reported engaging in physical activity with their child, namely cycling, hiking, stair climbing and badminton three to five times a week, whereas the parents of participant 2 reported not engaging in any physical activity as a family during the same period. Both would prefer an individualized (one-on-one) exercise intervention program.

Three of the five patients eligible for this pilot study declined to participate, either because they did not want to spend additional time in hospital to undergo non-essential tests or because of a lack of interest. Induction chemotherapy is the first and most aggressive phase of treatment, as it aims to destroy as many cancerous cells, called leukemic blasts, in order to achieve an initial remission.^37,38^ Children with ALL undergoing therapy are also known to be at risk for peripheral neuropathic pain, which typically occurs in the lower extremities^.^^7^ Cardiorespiratory test performance on a cycle ergometer may be limited by lower extremity pain and fatigue.^22^ Patients in the consolidation phase of chemotherapy have been shown to have significantly higher activity levels and spend significantly less time being sedentary than children in the induction phase, but they are still less active than healthy children.^33^ Being connected to multiple medical devices, room isolation or ward restriction may explain the low physical activity levels of children in the induction phase.^18,33^ An overprotective approach by parents and the medical team may also alter children's perception of their ability to engage in physical activity, inducing fear of overexertion and low self-efficacy,^14^ which may contribute to the low physical activity levels observed for these children. Participants in this pilot study mentioned that they appreciated the time taken to explain the risks associated with their participation in the project, the collaboration of the attending physician and medical team in the project, their interest in contributing to the research, the opportunity for parents to observe their child's physiological state throughout the chemotherapy treatment, and to be able to test their capacities. The fact that there were no adverse events related to cardiorespiratory and muscular testing for either participant also favored their participation in testing at their second visit.

Another objective of this pilot study was to observe whether participants experienced any physiological changes after the two testing sessions. Although many variables were not controlled during this pilot study (e.g., nutrition, sleep), the test results showed an overall improvement in participant 1's cardiorespiratory fitness, muscle tests scores, quality of life score, and overall physical activity level, whereas participant 2 experienced an overall decline. Rathe et al. showed that cardiotoxicity in children with ALL is observed during longitudinal cardiac follow-up.^10^ In fact, no cardiotoxicity was detected in either of the participants. Compared with T1, at T2, participant 1 achieved a higher maximum heart rate, cardiac output, maximal blood pressure, and minute ventilation as well as improved overall performance of her pulmonary and cardiac systems.^39,40^ Results presented in Figure 3 for participant 2 showed a regression in her physiological response to the test as she achieved 53% of her predicted $\dot{V}$ O_2_max based on her age, sex and weight at T2, compared with 64% at T1. It is possible that the additional doses of chemotherapeutic agents that participant 2 received during her additional three weeks of induction (induction B, part 2) contributed to her decreased performance at the T2 cardiorespiratory fitness tests. Another factor could be that participant 2 reported a reduction in physical activity level between T1 and T2 on the HAES questionnaire. It is also worth mentioning that participant 2 at T2 reported leg pain during the cardiorespiratory test and was receiving injections to treat her neuropathic pain.

As previously reported in the literature,^33^ the results of the participants’ cardiorespiratory and muscular function were below their predicted values for age and gender. A study by Kabak et al. showed that children with ALL scored below standard on tests of fine motor integration, manual dexterity, balance, running speed and agility, upper limb coordination and strength.^41^ This shows that the deconditioning in this population covers many physiological systems and various functional abilities.^41^ Brinksma et al. have shown that children with hematological cancers may be undernourished during the first three months of chemotherapy and thus have low fat-free mass compared to healthy children.^42^ Moreover, symptoms such as nausea and pain may limit food intake.^43^ Although nutritional status was not evaluated in this pilot study, it is possible that the participants’ caloric intake was not optimal at some point in the study. This could be one cause of the observed change in body composition in both participants, namely an average 40% increase in body fat, with minimal increase in lean body mass. Such changes in body composition have also been reported in the first year of children diagnosed with ALL.^44^ A similar study might consider asking participants, with parental support, to keep a diary of physical activity, sleep, hydration, nutrition and symptoms to gain insight into their physical changes and interpret these results. Hence, in this pilot study, the following elements may have contributed to the participants’ results: their illness, the chemotherapy treatment and its side effects, their motivation to perform during the tests, their nutritional status, their level of physical activity and a fear of overexertion. Although the sample size of this pilot study is too small to conclude that the disease risk-level and the associated chemotherapy regimen had an impact on the physiological changes observed in the two participants, it would be interesting to explore whether conclusions can be drawn from larger studies. Such information could help to better understand the physiological change and interventional needs of children with ALL, and whether the chemotherapy regimen has an influence on their capacities.

Several limitations that affected this pilot study must be recognized. First, a larger study with more participants of both sexes and all risk levels would be needed to replicate and consolidate its results. Such larger studies should include participants being treated with radiation therapy and bone marrow transplant in addition to the chemotherapy treatment regimen.^45^ A second limitation is that this study did not measure baseline values before chemotherapy (T0), because recruitment is difficult at the time of diagnosis largely due to parents’ emotional shock of learning that their child has cancer.^33^ Also, chemotherapy and admission occur very quickly, leaving no time for a long battery of physical tests. A third limitation is that it was not possible to perform both T2 tests in the same treatment cycle of consolidation 2 for both participants due to participant availability (a participant living outside the regional treatment center) and time constraint. A fourth limitation is that participants may not have reached their maximum aerobic capacity during the tests. Arguably, a more progressive protocol (for example, a ramped protocol) might have been better suited considering that participants would not have been subjected to abrupt changes in resistance. Gradual increase in resistance could have been better adapted to the demands of the test, allowing longer tests with more accurate values for $\dot{V}$ O_2_peak and possibly reaching a $\dot{V}$ O_2_max.^22,46^ As previously discussed, results of cardiorespiratory tests done on a cycle ergometer may be influenced by lower limb strength and endurance, notably due to the seated position throughout the test. The chemotherapeutic agent vincristine can provoke vincristine-induced peripheral neuropathy (VIPN) in children with ALL either through pain, motor and/or autonomic nervous system deficiency.^7^ Participant 2 may have suffered from VIPN syndrome in the lower limbs, which could have had a negative physiological impact on her ability to achieve maximum effort on the ergometer. Thus, cardiorespiratory test results for children with ALL may depend on the testing protocol and modality used (treadmill versus ergometer) as well as the presence or absence of VIPN at the time of testing, which should be noted and considered when interpreting results.

## Practical applications

In summary, assessment of maximal cardiorespiratory function during the first 10 months of chemotherapy was feasible and safe in this pilot study with two young adolescent girls with low- and high-risk B-cell ALL. This pilot study suggests early changes in physical abilities during chemotherapy treatment. Future studies with a larger number of female and male participants, including a wider range of age at disease diagnosis, as well as all the risk levels of the disease would be needed to replicate these preliminary findings and determine whether they are applicable to the population of children and adolescents with ALL.

## Appendix A

Questions of the Semi-Structured Interview with the Participant after T1

1. How did you find today's test session? Easy, medium or difficult?
2. Which test was the most tiring for you and why? (e.g., shortness of breath, leg pain)
3. Do you like to play sports or to do a physical activity?
4. Would you like to play sports with other children who have leukemia like you?
5. If yes to the previous question, please name an activity you'd like to do in a group.

Questions of the Semi-Structured Interview with the Parents after T1

1. What did you think of your child's performance today?
2. What was your impression of the tests your child took today? Did the tests seem easy, medium or difficult?
3. How would you describe your child's energy level at this moment?
4. What would you think of an adapted, individualized and progressive exercise program during treatment?
5. Were you physically active as a family before your child's diagnosis? If so, what kind of activity? At what intensity?
6. Was your child a member of a sports team or a regular participant in any sport before their diagnosis? If so, which sport? How many times a week? At what level? For how long?
7. What types of physical activity would you be comfortable doing with your children?

Questions of the Semi-Structured Interview with the Participant after T2

1. How did you find today's test session? Easy, medium or difficult?
2. Which test was the most tiring for you? Why? (e.g., shortness of breath, leg pain)
3. Was it easier or harder for you to do the tests today compared to the last time? Why or why not?
4. Have you done any physical activities or sports since the last time we saw each other? If so, can you tell me what you did?
5. Did you enjoy these physical activities?
6. How do you feel after doing a physical activity or sport?

Questions of the Semi-Structured Interview with the Parents after T2

1. What did you think of your child's performance today?
2. What was your impression of the tests your child took today? Did the tests seem easy, medium or difficult? How about a comparison with the first session?
3. How would you describe your child's energy level at the moment? Compared with the previous session?
4. Have you been active as a family or just with your child since our last meeting? If so, what kind of activity? At what intensity? How did it go for your child?
